# Microscopic Structural and Dynamic Features in Triphilic Room Temperature Ionic Liquids

**DOI:** 10.3389/fchem.2019.00285

**Published:** 2019-05-02

**Authors:** Fabrizio Lo Celso, Giovanni B. Appetecchi, Elisabetta Simonetti, Man Zhao, Edward W. Castner, Uwe Keiderling, Lorenzo Gontrani, Alessandro Triolo, Olga Russina

**Affiliations:** ^1^Dipartimento di Fisica e Chimica, Università di Palermo, Palermo, Italy; ^2^ENEA, Laboratory SSPT-PROMAS-MATPRO, Rome, Italy; ^3^Department of Chemistry and Chemical Biology, Rutgers University, The State University of New Jersey, Newark, NJ, United States; ^4^Soft Matter and Functional Materials, Helmholtz-Zentrum für Materialien und Energie GmbH, Berlin, Germany; ^5^Department of Chemistry, University of Rome Sapienza, Rome, Italy; ^6^Laboratorio Liquidi Ionici, Istituto Struttura della Materia, Consiglio Nazionale delle Ricerche (ISM-CNR), Rome, Italy

**Keywords:** fluorous tail, triphilic, ionic liquid, neutron scattering, molecular dynamics (MD)

## Abstract

Here we report a thorough investigation of the microscopic and mesoscopic structural organization in a series of triphilic fluorinated room temperature ionic liquids, namely [1-alkyl,3-methylimidazolium][(trifluoromethanesulfonyl)(nonafluorobutylsulfonyl)imide], with alkyl = ethyl, butyl, octyl ([C_n_mim][IM_14_], *n* = 2, 4, 8), based on the synergic exploitation of X-ray and Neutron Scattering and Molecular Dynamics simulations. This study reveals the strong complementarity between X-ray/neutron scattering in detecting the complex segregated morphology in these systems at mesoscopic spatial scales. The use of MD simulations delivering a very good agreement with experimental data allows us to gain a robust understanding of the segregated morphology. The structural scenario is completed with determination of dynamic properties accessing the diffusive behavior and a relaxation map is provided for [C_2_mim][IM_14_] and [C_8_mim][IM_14_], highlighting their natures as fragile glass formers.

## Introduction

Ionic liquids (ILs) constitute an interesting class of compounds, composed solely of ionic species and with a melting point below 100°C. Although some debate exists on their environmental compatibility, they are attracting a great deal of attention as they are considered potential replacements for noxious solvents and flammable electrolytes. Their fascinating chemical and physical properties can be widely modulated upon slight chemical modification of constituent ionic species. In the framework of their potential application in a variety of fields, the possibility of introducing a fluorous moiety into either the cation or the anion (e.g., by replacing an alkyl with an equivalent length perfluoroalkyl chain) is quite attractive, as it allows accessing further modulation of properties and performances. In these cases, the already appealing properties of conventional ILs, including non-flammability, negligible vapor pressure, high thermal, chemical and electrochemical stability, are maintained and can be further fine-tuned by introduction of fluorous moieties leading to high hydrophobicity, enhanced surface activity, interesting gas uptake properties etc. Fluorinated Ionic Liquids (FILs) show properties arising from both their fluorous and ionic natures, thus introducing interesting variations into the more conventional IL landscape. As both fluorinated compounds and ILs are considered neoteric solvents with the potential toimpact sustainable processes and green chemistry, it can be envisaged that their merging into the FILs technology will soon be of high impact (Merrigan et al., 2000; van den Broeke et al., 2002; Kim et al., 2004; Xue and Shreeve, 2005; Xue et al., 2006; Almantariotis et al., 2010, 2017; Smith et al., 2010; Kunze et al., 2011; Yoshida and Saito, 2011; Jeremias et al., 2013; Pereiro et al., 2013a, 2018; Weber et al., 2013; Hollóczki et al., 2015; Suarez et al., 2015; Wu et al., 2016; Rauber et al., 2017; Bastos et al., 2018).

**Scheme 1:**
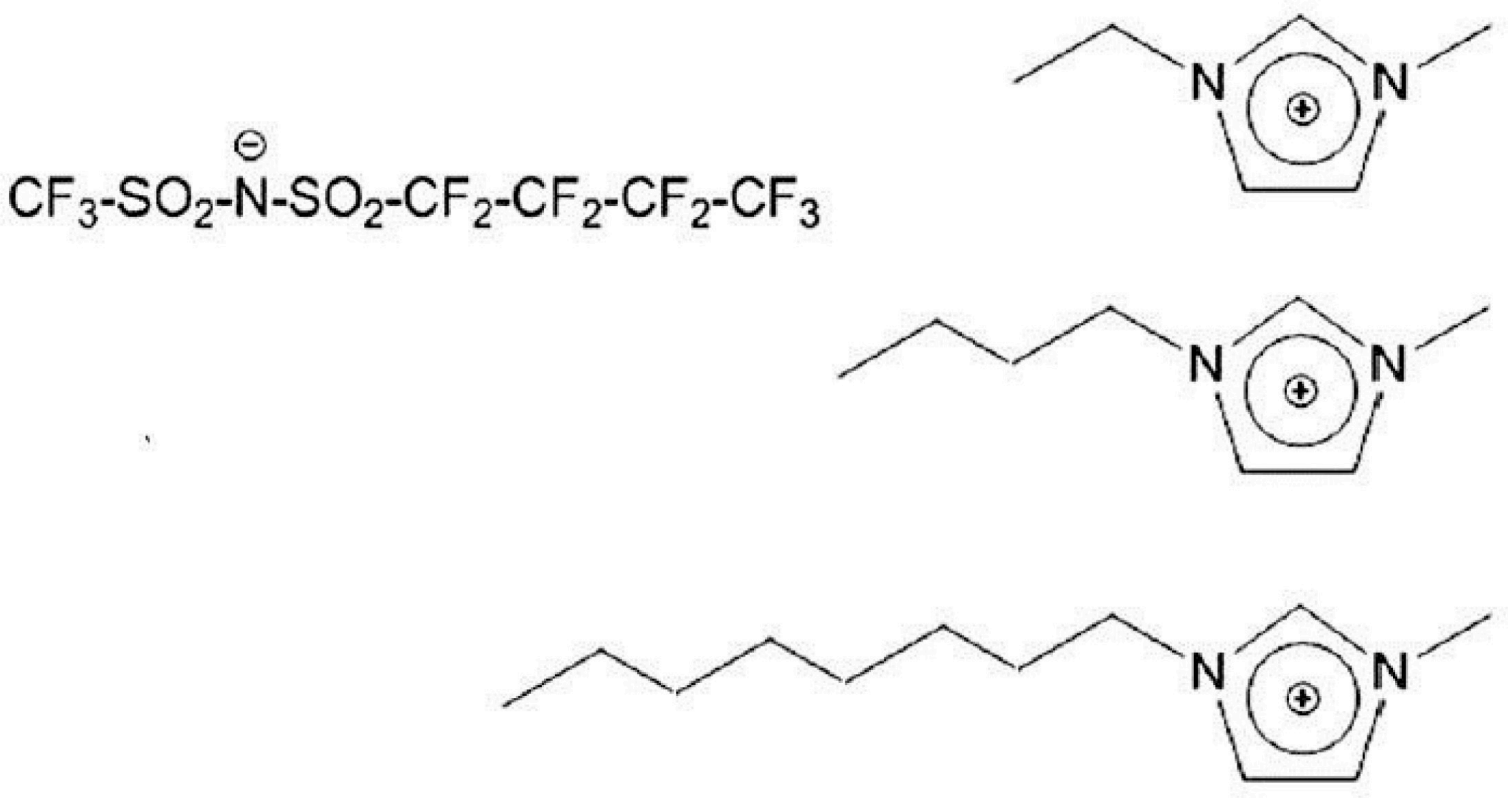
Description of chemical nature of the investigated ionic liquids. On the **(Left)**, the anion's structure is shown, while the **(Right)** part of the Scheme shows the different cations [1-alkyl-3-methylimidazolium, with alkyl = ethyl **(Top)**, butyl **(Middle)**, and octyl **(Bottom)**].

Among other peculiar features in FILs, their mesoscopic structural organization is quite interesting. It is well-known that alkyl chain bearing ILs are characterized by a distinct degree of nm-scale order, associated to the spatial segregation of apolar domains (viz. alkyl chains) with respect to a polar matrix (viz. anion and cation heads) (Urahata and Ribeiro, 2004; Wang and Voth, 2005, 2006; Canongia Lopes and Padua, 2006; Canongia Lopes et al., 2006; Pádua et al., 2007; Triolo et al., 2007; Almantariotis et al., 2010, 2017; Smith et al., 2010). Such a structural organization can be experimentally detected by means of X-ray and/or neutron scattering techniques, as it is fingerprinted by a distinct low momentum transfer (Q) peak (that is centered at a position reflecting the characteristic size of the segregated domain), as it was both experimentally and computationally found (Triolo et al., 2007, 2008, 2009; Atkin and Warr, 2008; Russina et al., 2009, 2012, 2017a,b; Hayes et al., 2011; Zheng et al., 2011; Li et al., 2012; Macchiagodena et al., 2012; Russina and Triolo, 2012, 2017; Song et al., 2012; Rocha et al., 2013). When introducing a fluorous moiety, as it is incompatible with both polar moieties and alkyl tails, it will tend to segregate into a third further kind of domain, where fluorous tails alone tend to cluster. This will lead to a characteristic mesoscopically organized morphology where three different, macroscopically incompatible, domains are forced to co-exist at the nm spatial scale: this led some of us to term such a kind of compounds as triphilic IL (Russina et al., 2013; Hollóczki et al., 2015). Such a scenario (Shen et al., 2012; Greaves et al., 2013; Pereiro et al., 2013b; Hettige et al., 2014; Brehm et al., 2015; Vieira et al., 2015; Ferreira et al., 2017; Shimizu et al., 2017) opens the way to simultaneous solvation of completely incompatible components into the homogeneous FIL, as each compound (e.g., a salt, an oil, and a fluorous compound) can be simultaneously dissolved into the same mesoscopically organized medium (Weiss et al., 2017; Tsurumaki and Ohno, 2018). In this contribution, we report on the structural properties of a series of 1-alkyl-3-methyimidazolium ([C_n_mim], where n indicates the number of carbon atoms along the side alkyl chain, with *n* = 2, 4, and 8) (trifluoromethanesulfonyl)(nonafluorobutylsulfonyl) imide ([IM_14_], 1 and 4 represent the number of carbon atoms on the two sides of the imide anion) [see e.g., (Johansson et al., 2010; Montanino et al., 2012; Jeremias et al., 2013; Russina et al., 2013, 2017a; Castiglione et al., 2014; Paolone et al., 2018)], at ambient conditions. Scheme 1 represents the chemical structure of the probed FILs.

## Experimental Details

### Samples Synthesis

The imidazolium-based, (trifluoromethanesulfonyl)(nonafluorobutylsulfonyl)imide ionic liquids were synthesized through a procedure developed at ENEA and described in detail elsewhere (Jeremias et al., 2013; Montanino et al., 2013). The chemicals, i.e., 1-methylimidazole (99 wt.%), bromoethane (99 wt.%), 1-bromobutane (99 wt.%), 1-bromooctane (99 wt.%), were purchased by Sigma-Aldrich and used as received. The sorbent materials, i.e., activated carbon (Darco-G60, Sigma-Aldrich) and alumina (acidic, Brockmann I, Sigma-Aldrich), were previously rinsed in deionized water according to a route reported elsewhere (Palumbo et al., 2017). Acidic (trifluoromethanesulfonyl)(nonafluorobutylsulfonyl)imide, HIM_14_ (59 wt.% solution in water), was purchased by 3M and used as received. Deionized water, used as the processing solvent, was obtained with a Millipore ion-exchange resin deionizer.

The imidazolium precursor was synthesized by reacting the 1-methylimidazole, previously dissolved in deionized water, with the appropriate amount of bromoethane (or 1-bromobutane or 1-bromooctane). A concentrated aqueous solution of imidazolium bromide was obtained, which was then purified through activated carbon and acidic alumina. The liquid fraction (clear and colorless) was separated from the solid one (sorbent materials) by vacuum filtering and separately collected. Successively, the solid phase was rinsed with deionized water to recover the precursor trapped through the purifying materials, obtaining a further aqueous solution (clear and colorless) which was unified to the previous one.

The imidazolium IM_14_ ionic liquids were obtained by reacting the precursor water solutions and the suitable amount of HIM_14_. The reaction (anion exchange) led to fast formation of hydrophobic ionic liquid and hydrophilic HBr (in water). After 1 h the mixing was interrupted and phase separation was allowed for a few minutes. The upper phase was mostly composed of water, HBr and HIM_14_ excess whereas the lower one was constituted of the ionic liquid with traces of water and HBr. After removal of the aqueous phase, the imidazolium IM_14_ ionic liquid was rinsed several times with deionized water to remove the HBr and HIM_14_ impurities. The ionic liquids were then housed in a vacuum rotary evaporator at 90°C to remove most of the water and, finally, vacuum dried at 120°C. The ionic liquid materials were stored in sealed glass tubes within a dry room.

The Li^+^ and Br^−^ content was checked by atomic absorption analysis (SpetcrAA mod. 220 atomic absorption Spectrometer) and X-ray fluorescence spectrometry (Shimadzu energy-dispersion EDX-720 spectrometer), respectively. Less than 2 ppm of lithium and bromide were detected in the ionic liquid samples.

### X-Ray Scattering

Small-Medium Angle X-ray scattering data were collected at the 11-ID-C beamline at the Argonne Photon Source (Ren, 2012). A monochromatic beam at a wavelength of 0.11165 A was used, with a beam size of 0.7 × 0.7 mm^2^. Samples were contained in a 2 mm diameter quartz capillary purchased from WJM-Berlin. Thirty consecutive data acquisitions were collected for a total acquisition time of 200 sec. Before merging the different data sets, care was taken that no radiation damage occurred to samples. Raw 2D data sets were integrated and transformed into intensities using the FIT2D software (Hammersley et al., 1996). The scattering structure factor S(Q) was calculated using the PDFgetX2 software (Qiu et al., 2004), correcting for background contribution, absorption, Compton scattering, and multiple scattering.

Additionally Small-Medium-Wide Angle X-ray scattering data were collected at beamline BL04B2 SPring-8 (Japan Synchrotron Radiation Research Institute, JASRI, Japan) (Kohara et al., 2001; Ohmura et al., 2009; Aoun et al., 2010). Measurements were conducted at ambient conditions. A monochromatized X-ray (61.6 keV) was obtained using a Si(220) monochromator. The observed scattering intensity of the X-ray was corrected for absorption, polarization, and incoherent scatterings (Cromer, 1969) to obtain coherent scattering intensities.

### Small Angle Neutron Scattering

Small Angle Neutron Scattering experiments (SANS) measurements on [C_n_mim][IM_14_] (*n* = 2, 4, 8) were performed at the small-angle scattering instrument V4 which is placed in the cold neutron guide of Helmholtz-Zentrum Berlin (HZB). The magnitude of the scattering vector is defined as Q = (4π/λ)(sinθ) with λ being the wavelength and 2θ the scattering angle. The measured neutron flux of the V4 is ~10^6^ cm^−2^.s^−1^ for the wavelength used, λ = 4.5 Å (Keiderling and Wiedenmann, 1995). The scattering data were obtained at one sample detector distance of 1.0 m, which yields a total momentum transfer range of 0.5 nm^−1^ < Q < 8 nm^−1^. For further information regarding the V4 instrument and its resolution the reader is referred to Keiderling and Wiedenmann (1995)and Gilles et al. (2000). The sample was placed into a circular quartz cuvette with inner spacing of 1 mm and placed in the beam for measurement. A Cd aperture of 13 mm was used for the scattering measurements. The 2D scattering data were reduced to a scattering curve [dΣ/dΩ(Q) vs. Q; hereinafter indicated as I(Q) vs. Q] by means of the BerSANS software. The raw data is then corrected for transmission, the quartz cell background scattering subtracted and converted to absolute units taking into account the scattering from water (Keiderling, 2002).

### Wide Angle Neutron Scattering

Neutron diffraction measurements on [C_2_mim][IM_14_] were performed on the SANDALS diffractometer at ISIS. The neutron wavelength range is 0.05–4.95 Å. Data were collected over the momentum transfer (Q) range between ca. 0.3 and 50 Å^−1^. The sample was contained in chemically inert, null scattering Ti_0.68_Zr_0.32_ flat cans (with size 3.5 × 3.5 × 1 mm^3^) sealed with Teflon O-rings. Measurements were also collected on the empty cell, so to properly subtract empty cell contribution. Furthermore, a measurement on a vanadium standard sample was also collected for data normalization purposes. Diffraction experiments were conducted at 298 K under vacuum. The diffraction pattern was measured for approximately 6 h at conditions of fully operating source. Data analysis was carried out using the GUDRUN software available at the facility that allows the application of (a) normalization to the incident flux, absorption and multiple scattering corrections, empty can subtraction, and normalization to absolute units by dividing the measured differential cross section by the scattering of a vanadium standard and (b) corrections for single atom scattering and hydrogen inelasticity effects.

### Diffusivity Measurements From PG-SE NMR

Self-diffusion coefficients for the anions and cations were measured using pulsed gradient spin-echo (PG-SE) NMR methods. Anionic diffusivities were measured from ^19^F NMR signals, and cationic diffusivities were obtained from ^1^H signals. The Diffusion-ordered bipolar pulse pair stimulated echo (DBPPSTE) pulse sequence was used (Wu et al., 1995). NMR samples were prepared from ionic liquids dried for 48 h on a Schlenk vacuum line, and prepared in an argon glovebox with water and oxygen levels below 0.1 and 0.4 ppm, respectively. Samples were prepared in 3 mm O.D. NMR tubes, capped in the glovebox, and flame-sealed immediately after removal from the glovebox. A Doty Scientific diffusion probe was installed on a 400 MHz Varian DirectDrive spectrometer for the PG-SE NMR measurements. PG-SE experiments had temperature uncertainties of ±1 K.

Additional details of our NMR diffusion protocols have been described previously (Liang et al., 2015; Wu et al., 2015, 2018).

### Viscosity Measurements

Temperature-dependent viscosity measurements of the ionic liquid samples were made using a Cambridge Viscosity ViscoLab 4100 instrument, as described previously (Funston et al., 2007; Mariani et al., 2017). Temperatures for the viscosity measurements were controlled to ±0.1 K by water flow from a Lauda Brinkmann RMT-6 recirculating chiller/heater.

### Dielectric Spectroscopy Measurements

Dielectric spectroscopy data were collected between 160 and 280 K in the range 2 10^−2^ – 3 10^6^ Hz, applying the gain-phase analysis technique with a Solartron SI-1260 analyser and a Novo-Control BDS 4000 spectrometer. The sample was sandwiched between two gold plated flat electrodes, with diameter of 20 mm and with a sample thickness of 50 μm. The temperature was controlled by a Quatro cryosystem.

### Computational Details

Molecular dynamic simulations were performed using the GROMACS 5.1.1 package (Van Der Spoel et al., 2005; Hess et al., 2008). Interactions were described using an all-atoms potential (Lopes and Padua, 2004; Shimizu et al., 2010). The simulations for [C_n_mim][IM_14_] ILs were performed using a cubic box of 900, 800, and 700 ion pairs for *n* = 2, 4, and 8, respectively; periodic boundary conditions were applied. Force field parameter files and initial configuration were created by DLPGEN software (Bernardes and Joseph, 2015); initial density was fixed 10% higher than the experimental one. A procedure of energy minimization has been performed starting from the initial configuration prior to the first NPT equilibration. The steepest descent algorithm implemented in Gromacs has been used and convergence has been obtained when every force on each atom would not exceed 1,000 kJ/mol/nm. Cutoff for the short range electrostatics and Van der Waals interaction were set to 1.0 nm. The equilibration procedure was done in several steps, starting from a series of NPT simulation at high temperatures and scaled partial charges, followed by lowering progressively the temperature and increasing the charges to their final value at 298,15 K and 1 bar, after a 15 ns run. This procedure was repeated two further times until an equilibrated system was obtained. After the equilibration phase, the system was run for a total of 50 ns for a production run, and then the trajectory of the last 5 ns was saved at a frequency of 1 ps, for calculation of the structural properties. The simulations were always checked vs. the experimental density and the energy profile. During the production runs for the temperature coupling, we used a velocity rescaling thermostat (Bussi et al., 2007) (with a time coupling constant of 0.1 ps), while for the pressure coupling, we used a Parrinello–Rahman barostat (Parrinello and Rahman, 1981) (1 ps for the relaxation constant). The Leap-Frog algorithm with a 1 fs time step was used for integrating the equations of motion. Cut-offs for the Lennard- Jones and real space part of the Coulombic interactions were set to 15 Å. For the electrostatic interactions, the Particle Mesh Ewald (PME) summation method (Darden et al., 1993; Essmann et al., 1995) was used, with an interpolation order of 6 and 0.08 nm of FFT grid spacing. Selected graphs were done using Matplotlib (Hunter, 2007). Weighted and partial structure factors were computed by using in-house developed software, accordingly to text book formulas as highlighted in Margulis's work (Kashyap et al., 2012), while selected pair correlation function, angular distribution function were obtained by TRAVIS (Brehm and Kirchner, 2011; Hollóczki et al., 2015). Ion aggregation analysis was performed using the AGGREGATES software (Bernardes, 2017).

## Results and Discussion

The chemical structure of [C_n_mim][IM_14_] samples with *n* = 2, 4, 8 that have been studied in this report is shown in Scheme 1. These samples differ only on the length of the side alkyl chain in the cation, the anion remaining unaltered. [C_2_mim][IM_14_] has been previously reported to be an homogeneous liquid at ambient conditions (Appetecchi et al., 2011). Analogously, [C_4_mim][IM_14_] and [C_8_mim][IM_14_] are liquid at ambient conditions and their synthesis and properties are here reported for the first time.

### Viscosity

The values for the viscosities for [C_2_mim][IM_14_] measured between 1.5 and 90°C spanned the range from 520 down to 12 cP. For [C_8_mim][IM_14_], the viscosities ranged from 1,011 to 28 cP over the temperature range from 3.7 to 70°C. As with all of our previous recorded viscosities for ionic liquids the data are best fit to the well-known Vogel-Fulcher-Tammann (VFT) equation in logarithmic form, given by: ln (η(T), cP) = ln (η_VFT_)+B/(T−T_o_); where η_VFT_, B, and T_o_ are fitting parameters, representing infinite temperature viscosity, an equivalent activation energy and a characteristic temperature, respectively.

The primary significance of these fits is that they provide an excellent representation of the data, permitting us to calculate an extrapolated viscosity for any arbitrary temperature in order to match the viscosity/temperature point for comparison with the diffusivities measured at different temperatures via PG-SE NMR. While the viscosity data does not fit as well to the Arrhenius equation as it does to the VFT equation, the Arrhenius fits are adequate for a qualitative discussion. The viscosity activation energies were found to be 37.2 kJ/mol for [C_2_mim][IM_14_] and 44.1 kJ/mol for [C_8_mim][IM_14_].

The fits of the viscosity data to the VFT and the Arrhenius equations are given in Figures S1, S2.

### Anion and Cation Diffusivities From PG-SE NMR

PG-SE NMR measurements were used to obtain the anionic and cationic diffusivities over the temperature range from 5 to 75°C for both [C_2_mim][IM_14_] and [C_8_mim][IM_14_]. Cationic diffusivities were observed to always larger than anionic diffusivities for both [C_2_mim][IM_14_] and [C_8_mim][IM_14_], though the difference is more pronounced for the former case. For the [C_2_mim][IM_14_] IL, the anion is significantly larger than the cation, while the opposite is true for [C_8_mim][IM_14_]. It is worth noting that the mass of the [IM_14_] anion (430 g/mol) is roughly four times that of the [C_2_mim] cation (*F*_w_ = 111 g/mol) and twice that of the [C_8_mim] cation (*F*_w_ = 195 g/mol). For [C_2_mim][IM_14_], the diffusivities range from 3.4 × 10^−12^ m^2^s^−1^ for the [IM_14_] anion at 5°C up to 1.1 × 10^−10^ m^2^s^−1^. For comparison, we note that the diffusivity of neat water is 1.26 × 10^−9^ m^2^s^−1^ at 4°C.

As is the case for viscosities, the ionic self-diffusivities can also be fit by both Arrhenius and VFT laws, with the latter always providing somewhat superior fit statistics, albeit at the cost of one additional fit parameter. Considering the activation energies from the Arrhenius fits, one finds that the E_a_ values for [C_2_mim][IM_14_] are slightly less than that for the viscosity: 32.5 and 35.4 kJ/mol for the cation and anion, respectively, as compared with 37.2 kJ/mol for the viscosity. The E_a_ values for [C_8_mim][IM_14_] self-diffusion coefficients are larger than [C_2_mim][IM_14_], but again, less than the E_a_ value for the viscosity: E_a_(cation) = 35.9 kJ/mol and E_a_(anion) = 37.7 kJ/mol, vs. 44.1 kJ/mol (viscosity).

Harris showed that hydrodynamic scaling is not exact for any molecular fluids considered, including ionic liquids (Harris, 2009). Specifically, the Stokes-Einstein prediction that the diffusivities should be proportional to the ratio of temperature over viscosity does not hold exactly. Rather, a scaled or fractional Stokes-Einstein law does hold, where D = (T/η)^α^, where α is an exponent between 0.9 and 1.0. By using the VFT fit parameters, we can calculate precise viscosities in order to make graphs of the ionic diffusivities vs. the ratio of T/η These plots of D vs. T/η are shown in Figure S3.

### Structure

**Figure 1:**
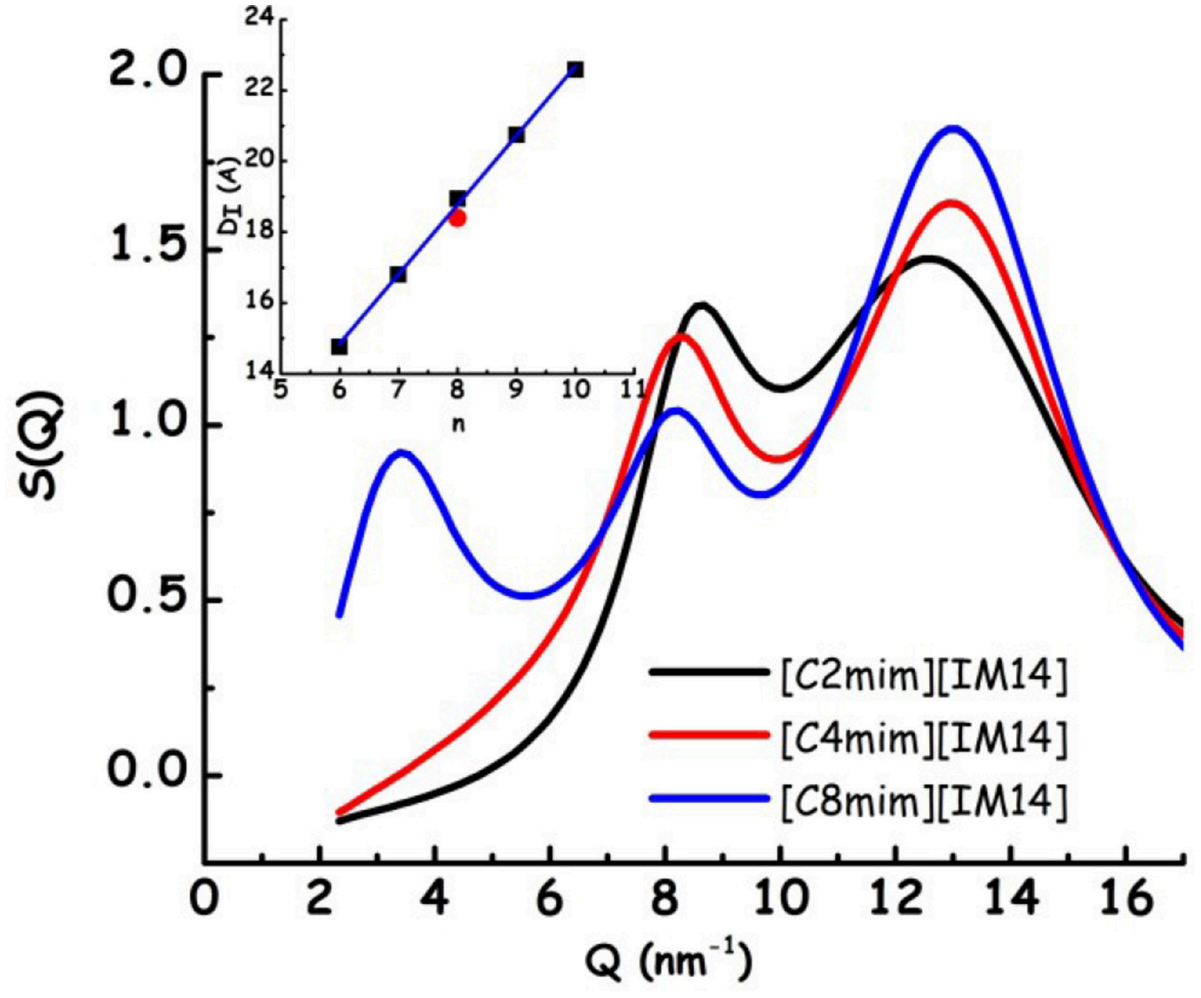
Experimental X-ray diffraction patterns from [C_n_mim][IM_14_] (*n* = 2, 4, 8) at ambient conditions. In the inset, the characteristic sizes of structural heterogeneities, obtained from 2π/Q_I_, where Q_I_ is the low Q peak in the diffraction pattern, are shown for [C_n_mim][IM_11_] (with *n* = 6–10) [from Russina et al. (2009)] (black symbols), together with the corresponding value from [C_8_mim][IM_14_] (present work, red symbol).

**Figure 2:**
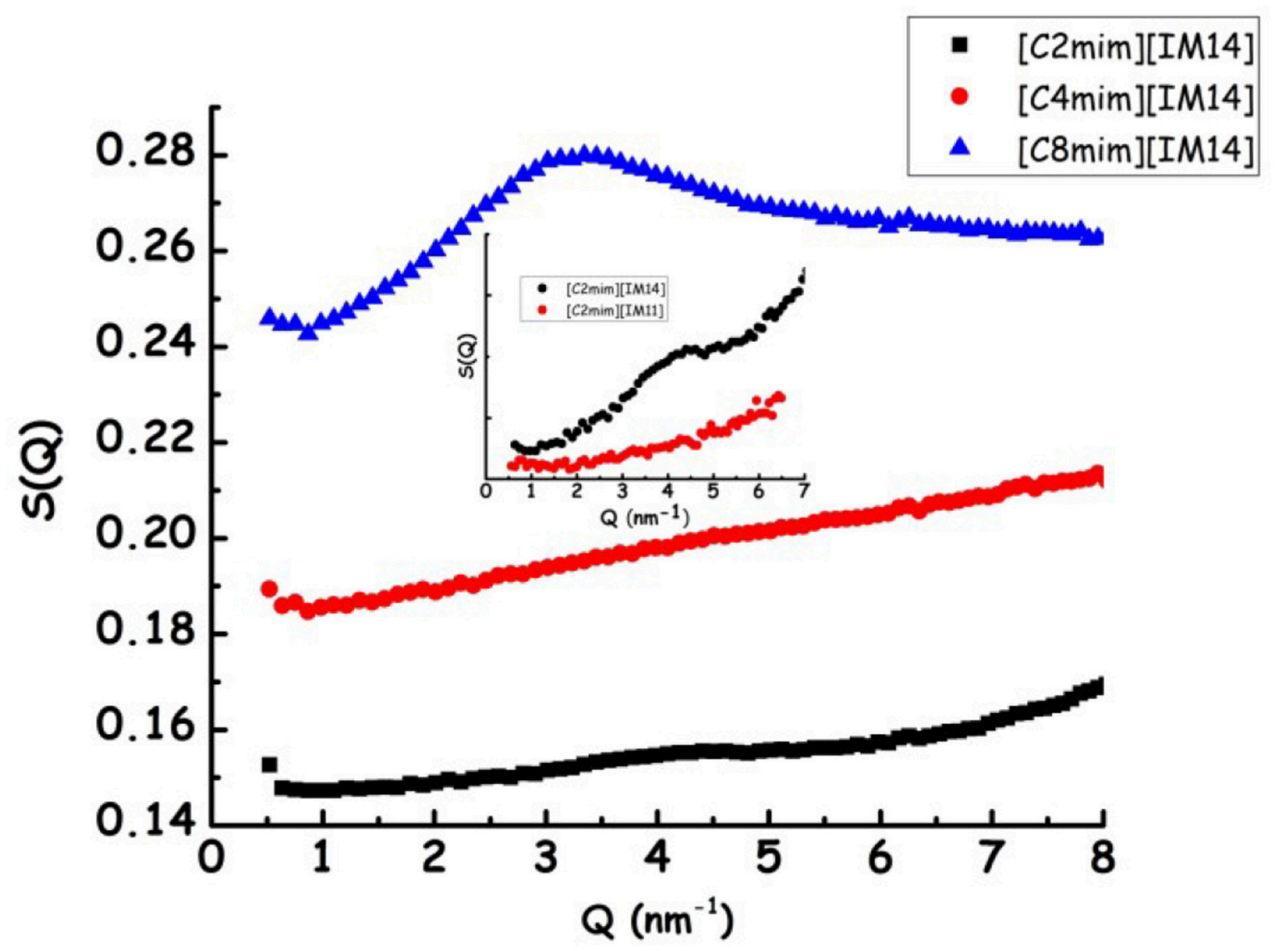
Experimental small angle neutron scattering data from [C_n_mim][IM_14_] (*n* = 2, 4, 8) at ambient conditions. In the inset a comparison between SANS data from [C_2_mim][IM_14_] and [C_2_mim][IM_11_] is shown.

In Figure 1, the X-ray diffraction patterns from the three samples are shown: in the reported Q range they are characterized by the presence of three peaks (Q_I−III_, in order of increasing Q position), that fingerprint the existence of different kinds of correlations (Q_I_ is better discernible in the case of [C_8_mim][IM_14_]). In particular, in order of decreasing Q position, these peaks have been shown to reflect the existence of: (a) adjacency, (b) charge, and (c) polar correlations (Annapureddy et al., 2010; Araque et al., 2015). It appears that, similarly to other cases, the peak that is most affected by increase in chain length is Q_I_ that reflects the establishment of polar-apolar correlations (Triolo et al., 2007, 2009; Russina and Triolo, 2012; Russina et al., 2012): this peak has negligible amplitude for short chain length and it grows in amplitude and shifts to lower Q value upon increasing n. Such a behavior has been reported for a multitude of ILs and reflects the progressive increase in size of the apolar domain embedded into the polar matrix that can be qualitatively quantified as D_I_~2π/Q_I_. In the inset of Figure 1, the characteristic domain sizes obtained for the case of [C_n_mim][IM_11_] (where [IM_11_] is the commonly used anion bistriflamide, [Tf_2_N]), reported by some of us in the past (Russina et al., 2009), are shown as a function of n. It can be observed that the corresponding quantity for the case of [C_8_mim][IM_14_] (for which Q_I_ peak position can be easily determined) is quite similar to the value reported for [C_8_mim][IM_11_], thus suggesting that X-ray scattering is a good reporter of the alkyl domain heterogeneities, whose size is only marginally affected by the different anion's nature. On the other hand, Figure 2 reports Small Angle Neutron Scattering (SANS) data from the same [C_n_mim][IM_14_] samples at ambient conditions, over the Q range up to 8 nm^−1^ (thus covering only the Q range where Q_I_ typically occurs, see Figure 1 for comparison). It clearly appears that while [C_8_mim][IM_14_] is characterized by a peak centered at ca. 3 nm^−1^ and [C_4_mim][IM_14_] shows a broad peaks superposition between 1 and 6 nm^−1^, [C_2_mim][IM_14_] is instead characterized by a distinct peak centered at ca. 4.5 nm^−1^. The latter peak has no analogous counterpart in the X-ray diffraction pattern shown in Figure 1. Moreover, the inset of Figure 2 showsa comparison between the SANS data from [C_2_mim][IM_14_] and [C_2_mim][IM_11_]: it is clear that while [C_2_mim][IM_11_] is essentially featureless in the probed Q range, on the other hand [C_2_mim][IM_14_] shows a distinct peak. The featureless pattern from [C_2_mim][IM_11_] is rationalized considering that the side alkyl chain in the cation (an ethyl moiety) is too short to deliver a clustering of chains segregating from the polar moieties, accordingly no polar-apolar separation occurs in this system. On the other hand the clear existence of low Q peak in the case of [C_2_mim][IM_14_] fingerprints the existence of mesoscopic structural heterogeneities that, due to contrast reasons, neutrons can detect. As recently reported in a series of papers (Shen et al., 2012; Russina et al., 2013; Hettige et al., 2014; Hollóczki et al., 2015; Lo Celso et al., 2017, 2018a,b), neutron scattering (and occasionally, X-ray scattering, *vide infra*) succeed in detecting the occurrence of fluorous tails clusters embedded into the charged matrix: the results presented in Figures 1, 2 confirm this observation, indicating that the perfluorobutyl chains in the [IM_14_] anions tend to associate into small fluorous domains and in the rest of the manuscript we will further explore this structural feature in the probed FILs.

**Figure 3:**
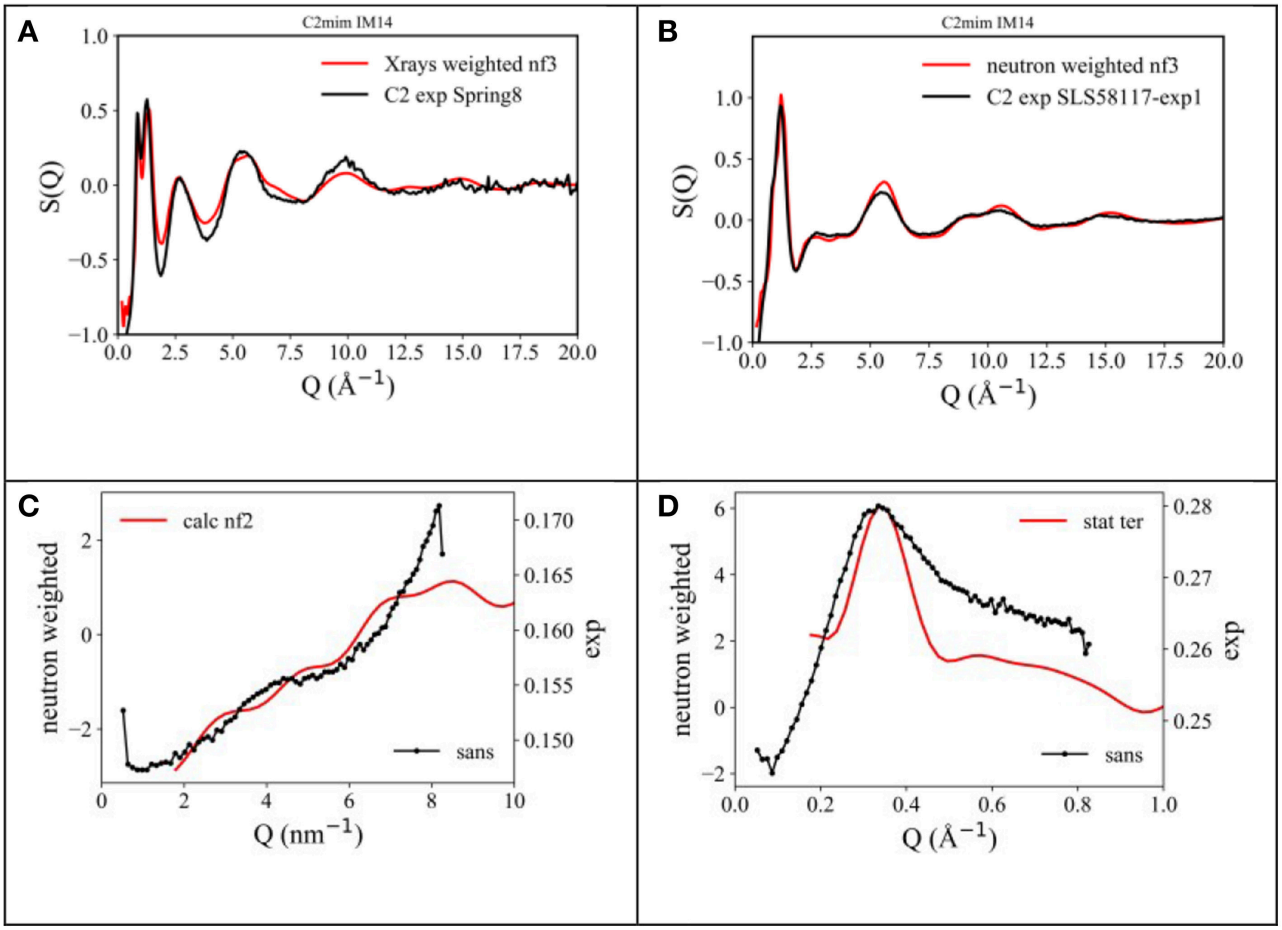
Comparison between **(A)** X-ray and **(B)** neutron diffraction data and the output from present MD simulations for the case of [C_2_mim][IM_14_] at ambient conditions. Also, comparison between experimental (black line) and un-normalized MD-computed (red line) SANS data from [C_n_mim][IM_14_], with **(C)** *n* = 4 and **(D)** *n* = 8, respectively, at ambient conditions.

In order to better address the issue of microscopic and mesoscopic structural organization in [C_n_mim][IM_14_] FILs, we undertook a series of detailed Molecular Dynamics simulations on these compounds, aiming to extract information at atomistic level. In the experimental part, we described the procedure and other details used for these simulations. In the case of [C_2_mim][IM_14_], the computed neutron and X-ray diffraction patterns have been directly compared with experimentally derived data sets. Figures 3A,B show such a comparison, while Figures 3C,D describe the comparison between the experimental SANS data and the corresponding computed quantities for the case of [C_n_mim][IM_14_], with *n* = 4 and 8: we consider the overall agreement between experimental and computational diffraction pattern very satisfactory. As a matter of fact, the simulations (along with the chosen interatomic potentials) succeed in accounting for all the structural features fingerprinted in the experimental diffraction patterns and this makes us confident that we can reliably interrogate the MD simulations to extract structural details at atomistic level.

**Figure 4:**
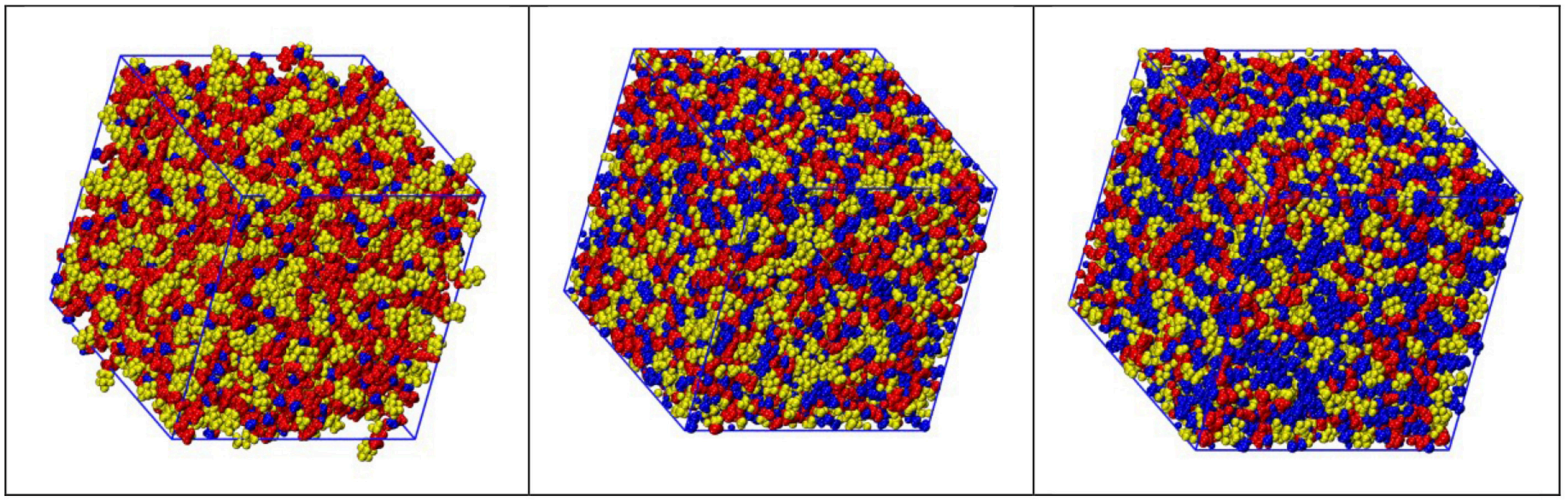
Representative snapshots derived from MD simulations from [C_n_mim][IM_14_], with *n* = 2, 4, 8 (left, center, and right, respectively), where color distinction of charged (red), alkyl (blue), and fluorous (yellow) moieties has been applied.

Figure 4 show representative snapshots of simulation boxes for the three different samples [C_n_mim][IM_14_] (*n* = 2, 4, 8), where polar moieties (imidazolium ring and SO_2_-N-SO_2_ moieties, belonging to cation and anion, respectively), alkyl tails and fluorous moieties are identified. A distinct mutual spatial segregation can be observed for these moieties, depending on the alkyl side chain length and we will better clarify this issue later on.

**Figure 5:**
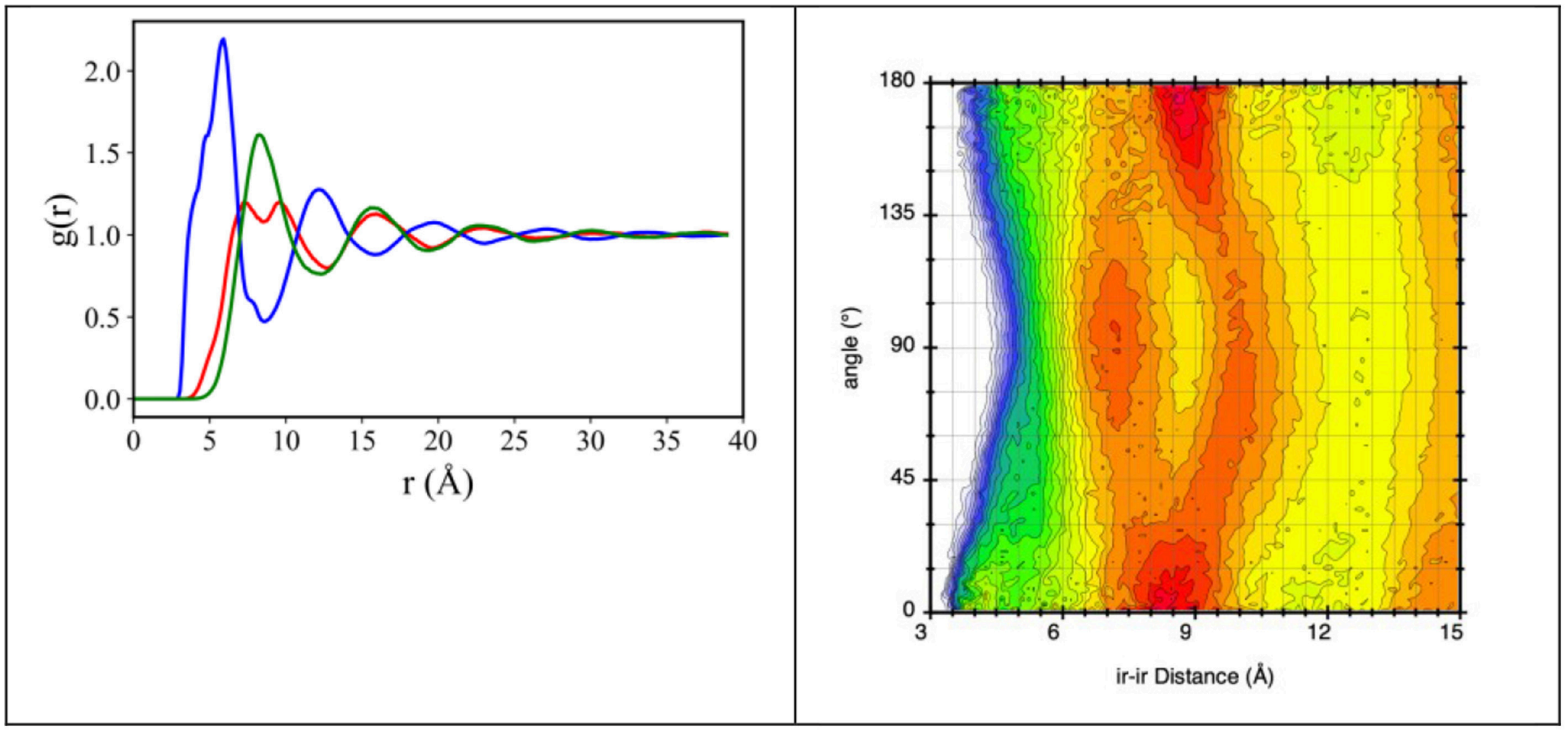
**(Left)** MD-derived pair distribution functions for ir-ir (red line), ir-N_anion_ (blue line), and N_anion_-N_anion_ (green line), where ir is the imidazolium ring center of mass and N_anion_ is the anion's nitrogen atom, from MD simulation of [C_2_mim][IM_14_] at ambient conditions. **(Right)** combined distribution function with abscissa the ir-ir distance and ordinate the angle between vector ir-ir and the vector normal to the imidazolium ring, from MD simulation of [C_2_mim][IM_14_] at ambient conditions. The plot explains the two maxima features occurring in the ir-ir pdf between 7 and 11 Å.

The characteristic alternation of oppositely charged layers around a chosen reference ion that is encountered in ILs, can be observed also in the present choice of compounds. Figure 5 shows the representative case of [C_2_mim][IM_14_] (different values of n lead to qualitatively similar results), where pair distribution functions (pdf) for: (a) center of mass of the imidazolium ring (ir) – ir; (b) ir – N_anion_ and (c) N_anion_ – N_anion_, N_anion_ being the nitrogen atom in the anion, are reported. It appears that cation-anion correlations are the shorter ones, occurring at ca. 4 Å, in agreement with the nature of coulombic interactions. Spatial correlations between similarly charged ions occur at a larger distance (ca. 10 Å) and such correlations are out of phase with opposite charges correlations (their maxima fall where opposite charge pdf has minima).

We notice that the cation-cation correlation (ir-ir) is characterized by a short distance correlation (red line in Figure 5 left) with two maxima at ca. 7 and 10 Å. In order to better rationalize this correlation, the combined distribution function (cdf), containing in abscissa the distance between neighbor imidazolium rings and in ordinate the angle (°) formed by vectors perpendicular to the imidazolium rings, has been evaluated and shown in Figure 5 right. We can then envisage that the observed splitting of the first solvation shell of imidazolium rings around a reference ring is the consequence of different packing of neighbor rings: the hottest lobes in Figure 5 right are found at ca. 8.2 Å for parallel or anti-parallel neighbor rings; however, non-negligible population is detected for perpendicular rings at distances of order of 7 and 10 Å.

**Figure 6:**
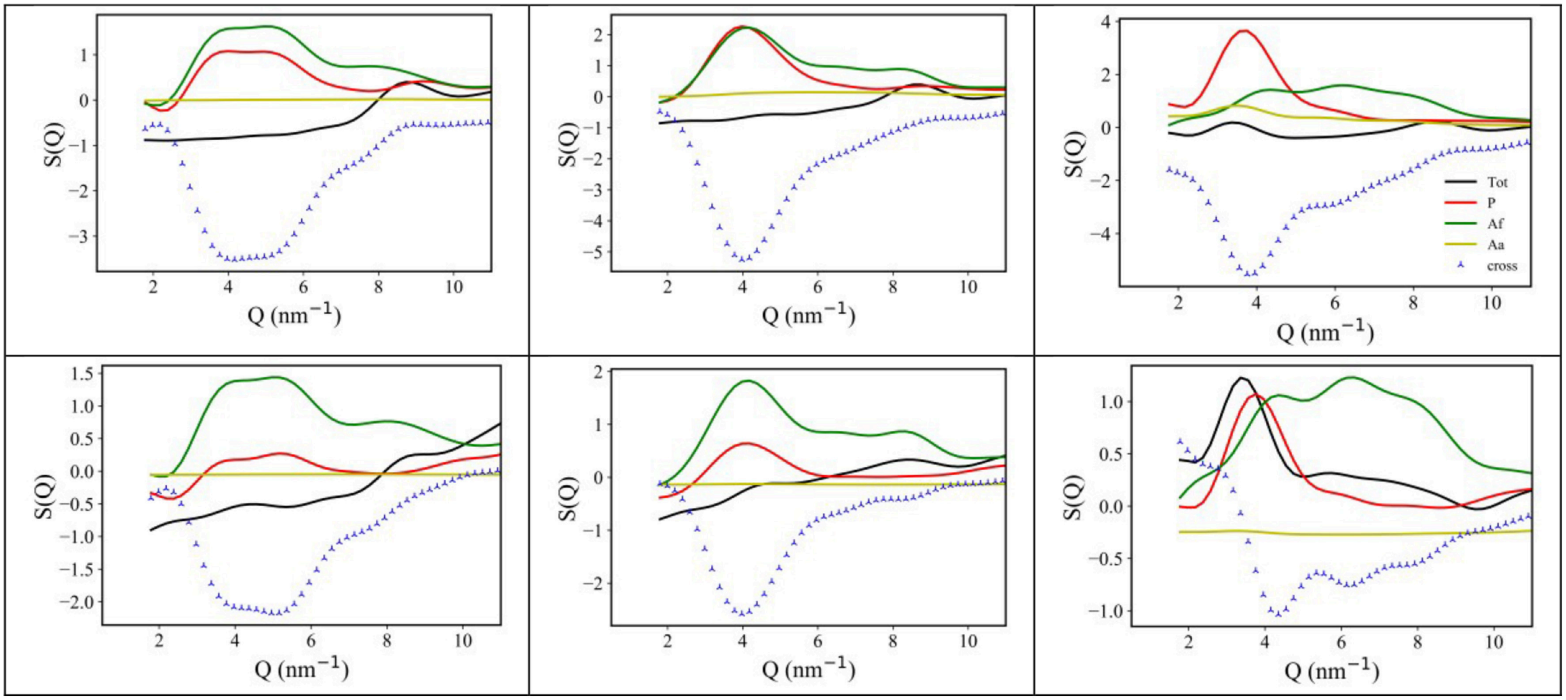
**(Top)** X-ray MD-computed diffraction patterns for [C_n_mim][IM_14_], with *n* = 2, 4, 8 (left, center, and right, respectively) and their decomposition into polar-polar (P), alkyl-alkyl (Aa), and fluorous-fluorous (Af-Af), total and cross terms; **(Bottom)** neutron MD-computed diffraction patterns for [C_n_mim][IM_14_], with *n* = 2, 4, 8 (left, center, and right, respectively) and their decomposition into polar-polar (P), alkyl-alkyl (Aa), and fluorous-fluorous (Af), total and cross terms.

**Figure 7:**
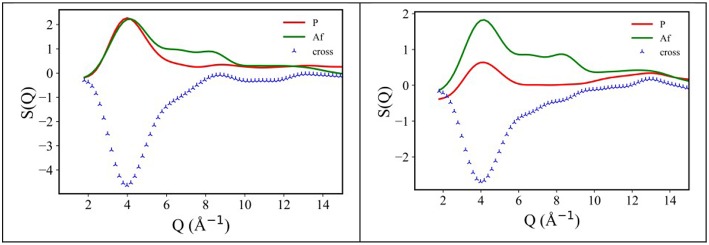
X-ray **(Left)** and neutron **(Right)** MD-computed diffraction patterns decomposition into polar-polar (P), fluorous-fluorous (Af), and polar-fluorous (cross) terms, for [C_4_mim][IM_14_] at ambient conditions.

Following an analysis approach introduced for the case of ILs by Margulis et al. (Annapureddy et al., 2010; Kashyap et al., 2012, 2013; Hettige et al., 2014; Araque et al., 2015), here we decompose the computed X-ray/neutron diffraction patterns in their components arising from structural correlations between polar and apolar moieties in the samples. While several examples exist of such a decomposition for the case of conventional ILs, where charged moieties and long alkyl tails represent the polar and apolar moieties, respectively, in the present case, similarly to recent analogous situations (Hettige et al., 2014), two different kinds of apolar moieties have been identified: namely, the alkyl and the fluorous tails. Such an approach stems from the quantitative interpretation of the visual situation depicted in Figure 4, where polar moieties as well as apolar alkyl and fluorous moieties tend to mutually exclude each other, thus introducing a complex triphilic structural scenario, with three different classes of nm-scale domains, whose size and mutual distribution determines the experimentally determined diffraction patterns. In the simple case of polar (P) and one (e.g., the alkyl moieties) apolar (A) domains, Margulis' group has shown that a characteristic decomposition can be achieved for the low Q peak, in terms of P-P, A-A, and A-P contributions, the two former terms manifesting into a positive amplitude peak and the latter into a negative amplitude one at the experimental peak position (Annapureddy et al., 2010; Kashyap et al., 2012, 2013; Hettige et al., 2014; Araque et al., 2015). In the case of two different apolar (Aa and Af, where the subscript a and f stand for alkyl and fluorous, respectively) domains coexisting with the polar (P) one, all the different self and distinct contributions should be considered to contribute to the overall experimental pattern (Hettige et al., 2014). Figure 6 report such decompositions of computed X-ray and neutron diffraction patterns; in the figures, the self-terms P-P (indicated therein as P), Aa-Aa (indicated therein as Aa), and Af-Af (indicated therein as Af) are shown together with the summation of the different distinct terms P-Aa, P-Af, Aa-Af (the combination of the latter terms being indicated as “cross”): the combination of these contributions leads to the total diffraction pattern. Overall, due to contrast reasons (contrast acts as a scale factor for each contribution and depends on either electron densities or scattering length densities for X-ray and neutron, respectively), the contribution of P/Aa alternation appears evident only in the case of [C_8_mim][IM_14_], where a positive amplitude peak is found for Aa-Aa and a negative amplitude one is found for P-Aa, both centered at a position Q_P/Aa_ ~3.5 nm^−1^, fingerprinting the existence of the P/Aa alternation and its characteristic size of the order of 2π/Q_P/Aa_~18 Å. The decomposition of both X-ray and neutron scattering allows detecting in a very clear way the structural alternation P/Af, between polar and fluorous domains. For example in Figure 7, the contributions P (Polar-Polar), Af (Af-Af), and cross (P-Af) to X-ray and neutron scattering are shown for [C_4_mim][IM_14_]. It appears that both in the case of X-ray and neutron scattering, peaks and anti-peaks related to both P-P and Af-Af and P-Af alternations, exist, respectively and fall at a position Q_P/Af_ ~4 nm^−1^. It is noteworthy that while in the case of X-ray scattering (Figure 7 left), the combination of these three contributions essentially vanishes, thus leading to no net contribution of the P/Af alternation to the total diffraction pattern, on the other hand, in the case of neutron scattering, the combination of the three terms leads to an overall non-zero contribution, that results into the low Q peak observed in the experimental neutron scattering data set (see Figures 1, 2).

**Figure 8:**
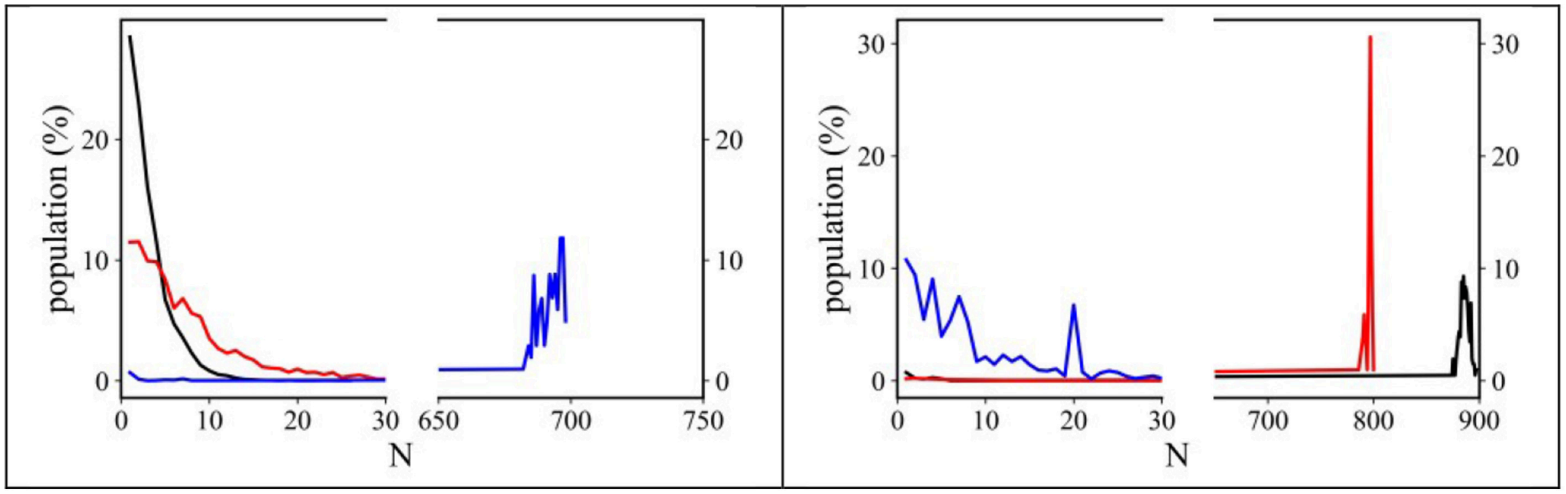
Normalized distribution functions for the alkyl **(Left)** and fluorous **(Right)** domain sizes from MD simulations of [C_n_mim][IM_14_] (*n* = 2, 4, 8, black, red, and blue, respectively) at ambient conditions. The highest value of the abscissa corresponds to the number of ion pairs contained in the simulation boxes.

An estimation of the degree of clustering of both alkyl and perfluoroalkyl tails can be also be obtained by monitoring the sizes of the clusters of such moieties. Using an approach developed by Bernardes et al. (2011, 2014), Shimizu et al. (2014) we analyzed our MD trajectories using the AGGREGATES software made available by Bernardes (2017) We considered CH_2_/CH_3_ moieties as belonging to an alkyl domain when neighbor carbon atoms were 5 Å apart, while we considered CF_2_/CF_3_ groups belonging to a fluorous domain when neighbor carbon atoms were 6 Å apart (Shimizu et al., 2017). Under these conditions, we monitored the tendency of both alkyl and fluorous chains to segregate into clusters. Figure 8 shows the trends observed for the dependence of (alkyl or fluorous) aggregates distribution sizes on the alkyl side chain length. It can be noticed that alkyl tails tend to form limited size clusters for the case of [C_n_mim][IM_14_] with *n* = 2 and 4 (clusters containing up to ~10 and ~30 methyl groups, respectively), while in [C_8_mim][IM_14_], octyl chains tend to percolate, as the size of aggregates reaches ~700 (representing the total number of anions present in the simulation box). Accordingly a gradual transition from finite size to percolating clusters is observed. In the case of anion's perfluorobutyl chains, [C_n_mim][IM_14_] with *n* = 2 and 4 show percolating nature of the chains clusters, while, in the case of [C_8_mim][IM_14_] a finite size distribution with maximum size of ~25 members is found, indicating also in this case the existence of a gradual transition from finite size to percolating clusters, though, with opposite trend with respect to *n*. When alkyl tails form finite size clusters, perfluorobutyl chain succeed in percolating the simulating box and, vice versa, when the latter chains form only finite clusters, it will be the alkyl chains that will percolate across the simulation box. Recently [C_n_mim]-based FILs with a fluorous anion have been simulated by Canongia- Lopes et al. (Pereiro et al., 2013b; Vieira et al., 2015; Ferreira et al., 2017; Shimizu et al., 2017). They found a behavior that is nice agreement with the present observations: while monitoring the aggregates detected in [C_n_mim][C_4_F_9_SO_3_], they also found that by increasing the side alkyl chain length of the cation, the alkyl domains tend to grow in size until they eventually percolate and this process is inverse in trend to the one observed for the fluorous domains sizes that instead decrease from the percolating regime to finite size clusters. Herein, on the basis of experimental results, supported by MD simulations, we further (Lo Celso et al., 2017, 2018b) validate this proposal.

### Main Relaxation Process

**Figure 9:**
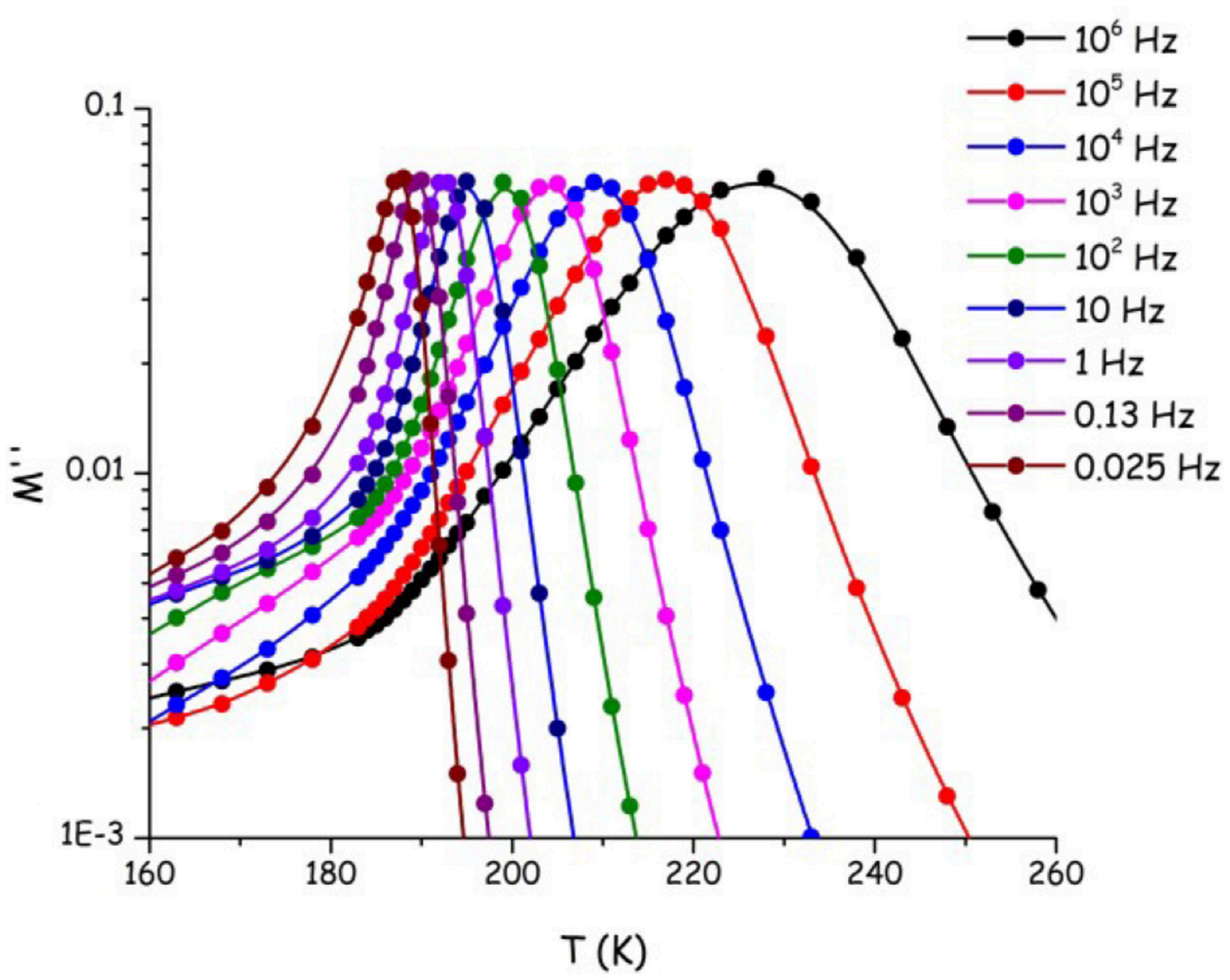
Imaginary part of the electric modulus (M^′′^) at isochronous conditions as a function of temperature, for [C_2_mim][IM_14_]. The temperature/frequency region associated to the main α process is shown.

**Figure 10:**
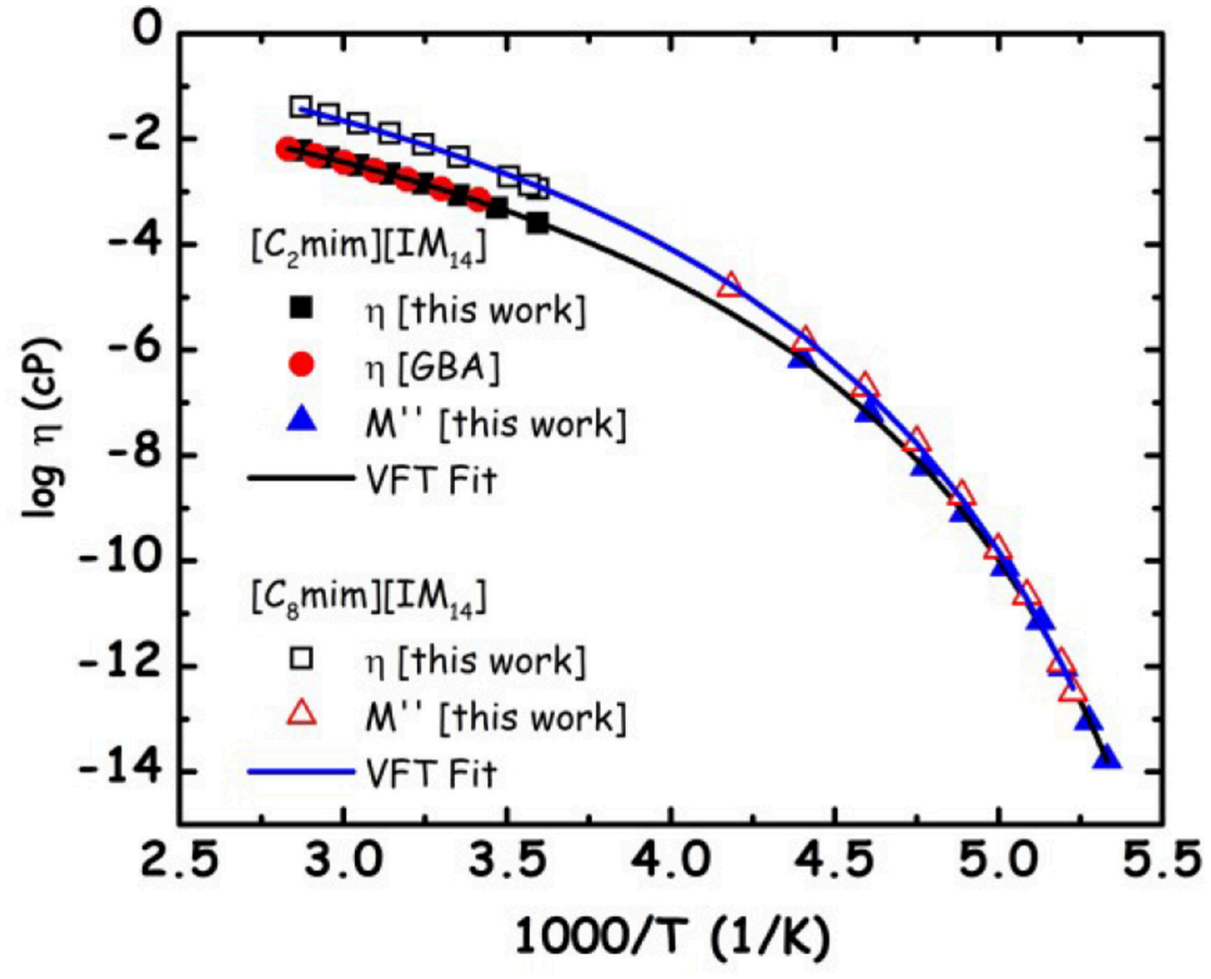
Relaxation map for the primary α-process for [C_2_mim][IM_14_] and [C_8_mim][IM_14_]. Viscosity data obtained in the present work and from the literature [GBA refers to Appetecchi et al. (2011)] and characteristic times from the imaginary part of the electrical modulus, M^′′^, are reported. The latter data sets have been vertically shifted in the logarithmic scale, taking advantage of the tTS that applies when dealing with different techniques probing the same relaxation process. Lines refer to the VFT fitting of the experimental data sets.

We aim now at providing a preliminary description of relaxation phenomena occurring in selected [C_n_mim][IM_14_] ILs, on the basis of experimental data. The nature of relaxation processes occurring in both [C_2_mim][IM_14_] and [C_8_mim][IM_14_] has been explored using a range of techniques. Figure 9 reports dielectric spectroscopy data collected for [C_2_mim][IM_14_] in the form of isochronal temperature dependence of the imaginary part of the electrical modulus, M^′′^. This formalism is commonly used when dealing with conducting samples, as it allows suppressing the contribution from conductivity and detecting the main α process. The latter process is generally associated to diffusive dynamics in glass formers and has equivalent signatures when monitored with other complementary techniques, such as viscosity or conductivity. In this case it is customary to account for the overall temperature dependence of the process by using the time/temperature superposition (tTS) that would provide a complete picture of the relaxation map of the chosen material. In Figure 10, we then report the relaxation map built up by the merging of different experimental data sets obtained for the case of [C_2_mim][IM_14_] as well as [C_8_mim][IM_14_]. While a more extended interpretation of these experimental results will be presented elsewhere, here we compare data arising from the above mentioned dielectric spectroscopy experiment, from previously published (Appetecchi et al., 2011) and new (this work) viscosity experiments. Using the tTS approach, data referring to the viscosities of the two ILs are combined with the characteristic times extracted from the M^′′^ data sets (once properly vertically scaled) and are jointly modeled in terms of a Vogel-Fulcher-Tamman (VFT) temperature dependence (Fulcher, 1925; Tammann and Hesse, 1926): log x = log x_o_+B/(T−T_o_), where x refers to a given quantity (e.g., viscosity, relaxation time etc.), x_o_, B, and T_o_ are fitting parameters, accounting for the temperature dependence of the observed property. The VFT formalism nicely accounts for the divergence of diffusion-related quantities (viscosity and characteristic time for the main α-process in the dielectric spectrum), when approaching the glass transition. The dielectric data sets indicate that characteristic times of 100 s are reached at 187 and 190 K for [C_2_mim][IM_14_] and [C_8_mim][IM_14_], respectively. These are then considered to be the dielectric glass transitions (T_g,diel_) for these compounds. On the basis of the VFT fitting parameters and the obtained T_g,diel_'s, one can derive the corresponding fragility indexes (Angell, 1985, 1991; Böhmer and Angell, 1992; Böhmer et al., 1993), after: m = B/ln(10) T_g_/(T_g_−T_o_)^2^: we obtain then values of 84 and 76 for [C_2_mim][IM_14_]'s and [C_8_mim][IM_14_]'s fragilities, respectively. These are relatively large values for the fragility index and reflect some degree of fragility in this class of compounds [the larger m, the more fragile is the compound, according to the definition proposed by Angell (1985, 1991), Böhmer and Angell (1992), Böhmer et al. (1993). The trend toward decreasing fragility (m) upon increasing alkyl chain length in ILs has been shown elsewhere, e.g., Sippel et al. (2015), Leys et al. (2008), Tao et al. (2017), where m values for a series of [C_n_mim]-based ILs with [BF_4_] and [PF_6_] anions have been reported and its relevance for energy applications highlighted.

## Conclusion

Here we have reported a detailed characterization of the triphilic nature of morphology in a series of imidazolium based ILs bearing a fluorinated anion. In particular samples with an alkyl chain in the cation and a perfluorobutyl moiety in the anion have been investigated. The synergic exploitation of complementary scattering techniques and Molecular Dynamics simulations allows extracting precise structural information at atomistic level. The complementarity of X-ray and neutron scattering is crucial to assess a specific feature of this class of FILs: It is clear that due to contrast reasons, X-ray scattering can detect only the existence of segregated clusters formed by alkyl chains. Neutron scattering instead allows detection of the existence of both alkyl and perfluoro alkyl segregated domains. Our careful MD simulations were able to accurately reproduce this behavior as well as the whole experimental data sets. Complementary approaches based on either diffraction pattern decomposition or aggregates analysis allowed to quantify the extent of (alkyl or perfluoroalkyl) chains clustering: upon increasing the alkyl chain length a complex transition from small to percolating alkyl chain clusters is observed; such a transition develops simultaneously with the inverse transition from percolating to small perfluoroalkyl chains clusters.

We also accounted for experimental determination of the diffusion related relaxation process for the case of [C_2_mim][IM_14_] and [C_8_mim][IM_14_], by determining their relaxation map and the fragility index, indicating their fragile behavior.

## Author Contributions

FLC developed MD simulations. GA and ES synthetized and characterized samples. MZ and EC executed viscosity and NMR measurements. UK, AT, and OR executed SANS experiments. AT and OR executed x-ray/neutron experiments. FLC, GA, EC, AT, and OR discussed the results. FLC, AT, and OR wrote the manuscript. All the authors revised the manuscript.

### Conflict of Interest Statement

The authors declare that the research was conducted in the absence of any commercial or financial relationships that could be construed as a potential conflict of interest. The handling editor declared a shared affiliation, though no other collaboration, with one of the authors FLC.
