# Supplementary material for: Microscopic Structural and Dynamic Features in Triphilic Room Temperature Ionic Liquids
Source: Front Chem. 2019 May 2;7:285. doi: 10.3389/fchem.2019.00285 (PMC6507529; doi:10.3389/fchem.2019.00285)
Supplement: Supplementary file 1 [file Data_Sheet_1.PDF]

Microscopic structural and dynamic features in triphilic room temperature ionic liquids.

F. Lo Celso[a], G. B. Appetecchi[b], E. Simonetti [b], M. Zhao[c], Edward W. Castner, Jr.[c], U. Keiderling[d], L. Gontrani[e], A. Triolo[f,*] and O. Russina[e,*]

[a] Dipartimento di Fisica e Chimica, viale delle Scienze, ed. 17, 90128 Palermo, Italy.

[b.] ENEA, Laboratory SSPT-PROMAS-MATPRO, Rome 00123, Italy.

[c.] Department of Chemistry and Chemical Biology, Rutgers, The State University of New Jersey, United States.

[d.] Soft Matter and Functional Materials, Helmholtz-Zentrum für Materialien und Energie GmbH, Berlin, Germany

[e.] Department of Chemistry, University of Rome Sapienza, Rome, Italy

[f.] Laboratorio Liquidi Ionici, Istituto Struttura della Materia, Consiglio Nazionale delle Ricerche, (ISM-CNR) Rome, Italy

[*] triolo@ism.cnr.it (A.T.); olga.russina@uniroma1.it (O.R.)

Supplementary Information

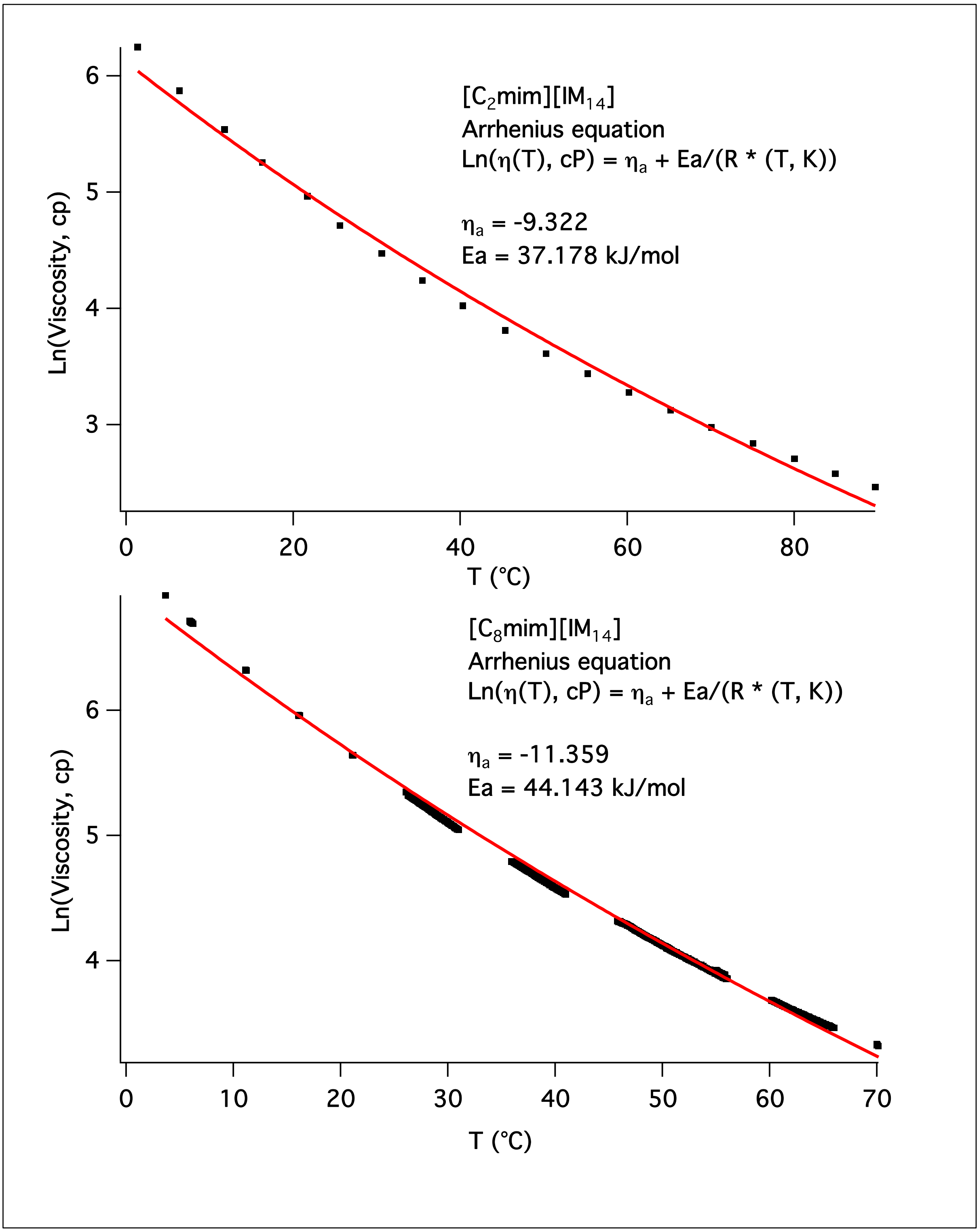

Figure SS1. Fit of viscosity data for $[C_2mim][IM_{14}]$ and $[C_8mim][IM_{14}]$, using the Arrhenius equation.

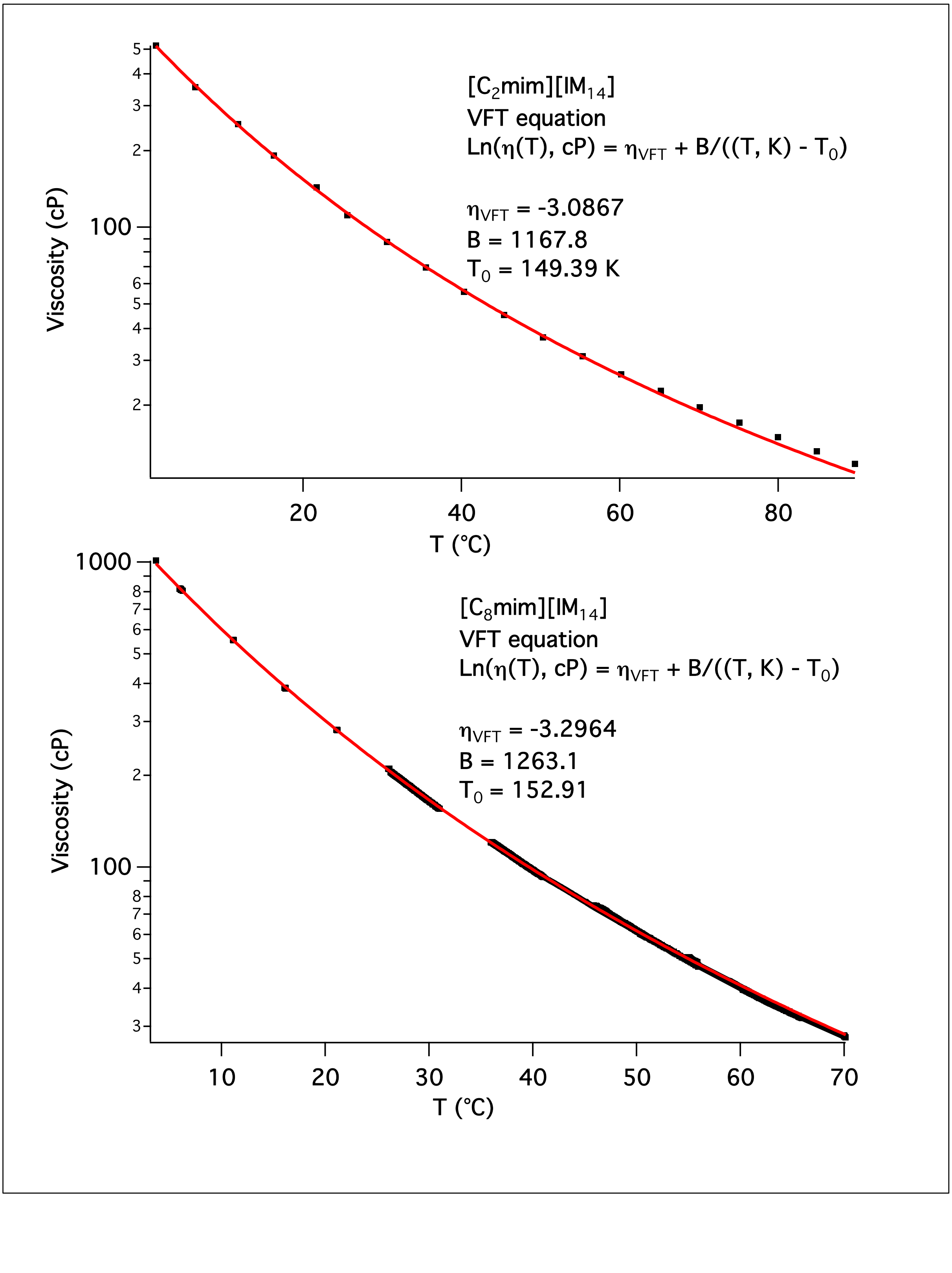

Figure SS2. Fit of viscosity data for $[C_2mim][IM_{14}]$ and $[C_8mim][IM_{14}]$, using the VFT equation.

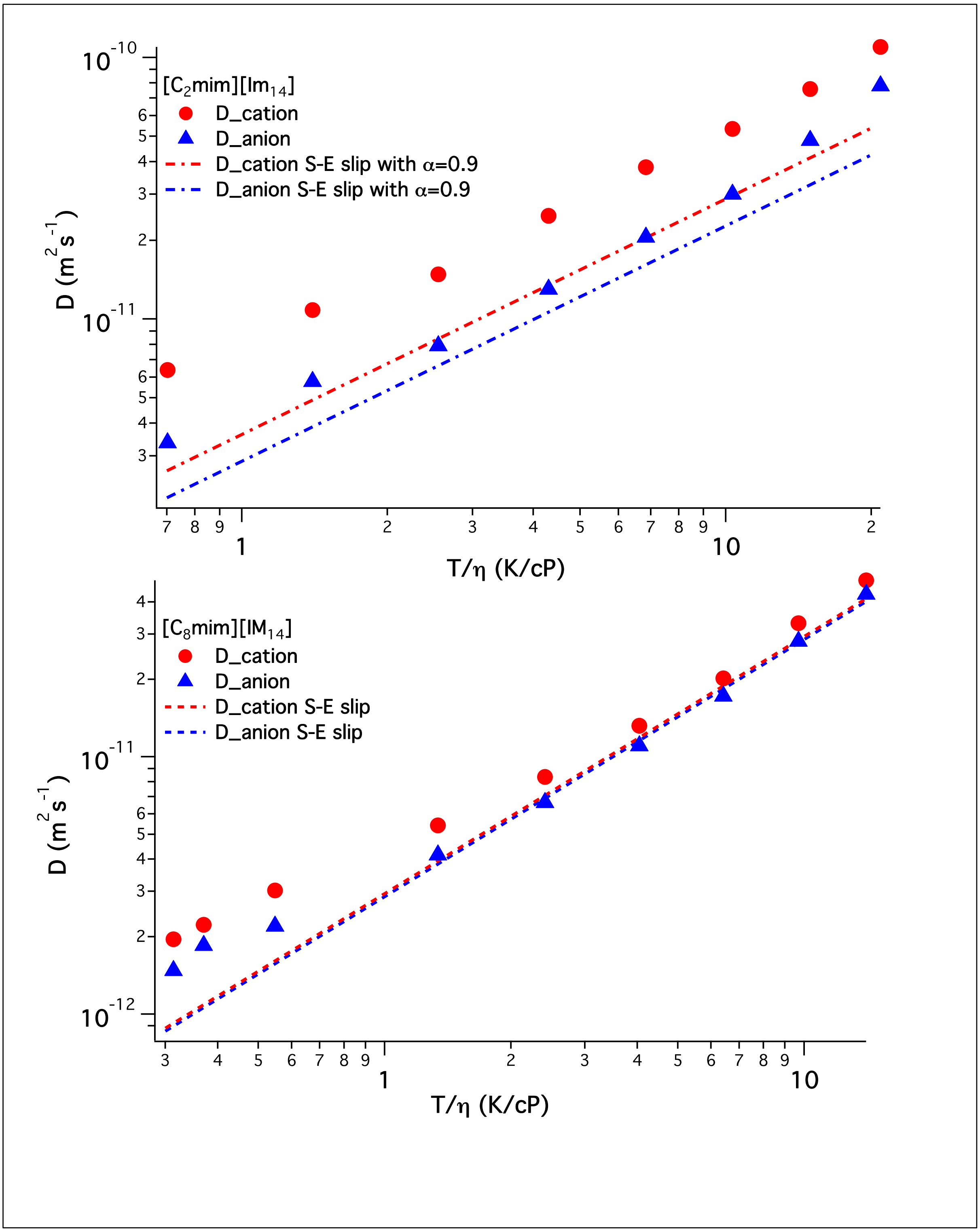

Figure SS3. Diffusion coefficients (for cation and anion) vs T/η for [C_2mim][IM_{14}] and [C_8mim][IM_{14}]. The lines show the fractional Stokes-Einstein trend fitted to the data.

*.gen files used for the present simualtions

Fc4c1si.gen

24

1	CBT	0.0000000000E+00	0.0000000000E+00	0.0000000000E+00
2	FBT	0.1332000000E+01	0.0000000000E+00	0.0000000000E+00
3	FBT	-.3916617132E+00	0.1273116296E+01	0.0000000000E+00
4	FBT	-.3916617132E+00	-.6365581479E+00	-.1102551054E+01
5	SBT	-.6751467250E+00	-.8439936166E+00	0.1461839825E+01
6	NBT	-.2155419900E+01	-.6922369421E+00	0.1272733401E+01
7	OBT	-.1670062570E+00	-.2186337513E+01	0.1312993833E+01
8	OBT	-.1099626776E+00	-.8604376552E-01	0.2551944415E+01
9	SBT	-.2925936568E+01	-.1734926099E+01	0.5183439879E+00
10	OBT	-.1946976107E+01	-.2234792704E+01	-.4165457345E+00
11	OBT	-.4003045889E+01	-.9709348144E+00	-.6337916632E-01
12	CB1	-.3413431048E+01	-.2894718781E+01	0.1830728865E+01
13	FBT	-.4150941413E+01	-.2210653132E+01	0.2703859238E+01
14	FBT	-.2292166429E+01	-.3353234697E+01	0.2384581562E+01
15	CFS	-.4215610893E+01	-.4014069875E+01	0.1166348233E+01
16	FCT	-.3415860447E+01	-.4590577969E+01	0.2706576381E+00
17	FCT	-.5274635431E+01	-.3447996404E+01	0.5899353143E+00
18	CFS	-.4625610376E+01	-.4989495074E+01	0.2270108672E+01
19	FCT	-.5363120741E+01	-.4305429424E+01	0.3143239045E+01
20	FCT	-.3504345757E+01	-.5448010989E+01	0.2823961369E+01
21	CFT	-.5427790222E+01	-.6108846168E+01	0.1605728040E+01
22	FCT	-.4628039776E+01	-.6685354262E+01	0.7100374448E+00
23	FCT	-.6486814760E+01	-.5542772697E+01	0.1029315121E+01
24	FCT	-.5784964395E+01	-.6958595262E+01	0.2567277356E+01

```
#
#        FBT  OBT      OBT  FBT  FCT  FCT  FCT
#         |   |         |   |    |    |    |
#         |   |         |   |    |    |    |
#     FBT--CB1--SBT--NBT--SBT--CB1--CFS--CFS--CFT--FCT
#         |   |         |   |    |    |    |
#         |   |         |   |    |    |    |
#        FBT  OBT      OBT  FBT  FCT  FCT  FCT
#
#
# Force field data from Cl&P:
# K. Shimizu, D. Almantariotis, M.F.C. Gomes
# A.A.H. Padua J.N. Canongia Lopes
# J Phys Chem B, 2010, 114, 3592â€“3600.


ATOM      1  C1          0.000   0.000   0.000
ATOM      2  F2          1.332   0.000   0.000
ATOM      3  F3         -0.392   1.273   0.000
ATOM      4  F4         -0.392  -0.637  -1.103
ATOM      5  S5         -0.675  -0.844   1.462
ATOM      6  N6         -2.155  -0.692   1.273
ATOM      7  O7         -0.167  -2.186   1.313
ATOM      8  O8         -0.110  -0.086   2.552
ATOM      9  S9         -2.926  -1.735   0.518
ATOM     10  O10        -1.947  -2.235  -0.417
ATOM     11  O11        -4.003  -0.971  -0.063
ATOM     12  C12        -3.413  -2.895   1.831
```

ATOM	13	F13	-4.151	-2.211	2.704
ATOM	14	F14	-2.292	-3.353	2.385
ATOM	15	C15	-4.216	-4.014	1.166
ATOM	16	F16	-3.416	-4.591	0.271
ATOM	17	F17	-5.275	-3.448	0.590
ATOM	18	C18	-4.626	-4.989	2.270
ATOM	19	F19	-5.363	-4.305	3.143
ATOM	20	F20	-3.504	-5.448	2.824
ATOM	21	C21	-5.428	-6.109	1.606
ATOM	22	F22	-4.628	-6.685	0.710
ATOM	23	F23	-6.487	-5.543	1.029
ATOM	24	F24	-5.785	-6.959	2.567

C8mim.gen

37

1	NA	0.0000000000E+00	0.0000000000E+00	0.0000000000E+00
2	CR	0.0000000000E+00	0.0000000000E+00	0.1315000000E+01
3	NA	0.1237000000E+01	0.0000000000E+00	0.1760000000E+01
4	CW	0.2082000000E+01	0.0000000000E+00	0.6720000000E+00
5	CW	0.1311000000E+01	0.0000000000E+00	-.4260000000E+00
6	C1	-.1180000000E+01	-.0000000000E+00	-.8700000000E+00
7	HCR	-.8840000000E+00	0.0000000000E+00	0.1936000000E+01
8	C1	0.1654000000E+01	0.0000000000E+00	0.3166000000E+01
9	HCW	0.3162000000E+01	0.0000000000E+00	0.6970000000E+00
10	HCW	0.1653000000E+01	0.0000000000E+00	-.1450000000E+01
11	H1	-.1168000000E+01	-.8900000000E+00	-.1500000000E+01
12	H1	-.2082000000E+01	-.0000000000E+00	-.2590000000E+00
13	H1	-.1168000000E+01	0.8900000000E+00	-.1500000000E+01
14	C2	0.2857000000E+01	0.9260000000E+00	0.3342000000E+01
15	H1	0.8310000000E+00	0.3510000000E+00	0.3789000000E+01
16	H1	0.1928000000E+01	-.1012000000E+01	0.3464000000E+01
17	CS	0.3291000000E+01	0.9260000000E+00	0.4808000000E+01
18	HC	0.3680000000E+01	0.5750000000E+00	0.2720000000E+01
19	HC	0.2584000000E+01	0.1939000000E+01	0.3045000000E+01
20	HC	0.2469000000E+01	0.1278000000E+01	0.5431000000E+01
21	HC	0.3566000000E+01	-.8500000000E-01	0.5106000000E+01
22	CS	0.4503000000E+01	0.1859000000E+01	0.4985000000E+01
23	HC	0.5318000000E+01	0.1501000000E+01	0.4391000000E+01
24	HC	0.4241000000E+01	0.2848000000E+01	0.4672000000E+01
25	CS	0.4920000000E+01	0.1884000000E+01	0.6467000000E+01
26	HC	0.5173000000E+01	0.8930000000E+00	0.6783000000E+01

```
  27 HC     0.4108000000E+01   0.2250000000E+01   0.7060000000E+01
  28 CS     0.6141000000E+01   0.2806000000E+01   0.6642000000E+01
  29 HC     0.6954000000E+01   0.2438000000E+01   0.6052000000E+01
  30 HC     0.5888000000E+01   0.3796000000E+01   0.6324000000E+01
  31 CS     0.6555000000E+01   0.2834000000E+01   0.8125000000E+01
  32 HC     0.6805000000E+01   0.1844000000E+01   0.8445000000E+01
  33 HC     0.5742000000E+01   0.3204000000E+01   0.8716000000E+01
  34 CT     0.7777000000E+01   0.3755000000E+01   0.8301000000E+01
  35 HC     0.8064000000E+01   0.3774000000E+01   0.9331000000E+01
  36 HC     0.8590000000E+01   0.3386000000E+01   0.7711000000E+01
  37 HC     0.7526000000E+01   0.4745000000E+01   0.7982000000E+01

#              HCR
#               |
#    H1        CR       H1  HC   / HC \     HC
#      \     / __ \    |  |  | |  |   /
#   H1--C1--NA  /  \  NA--C1--C2--|--CS--|--CE--HC
#      /    \ \__/ /   |  |  | |  |   \
#    H1      CW -- CW    H1  HC   \ HC /     HC
#           /     \               5
#         HCW     HCW
#
#
# Force field data from Cl&P:
#
# J.N. Canongia Lopes, J. Deschamps, A.A.H. Padua
# J Phys Chem B, 2004, 108, 2038-2047.
#
```

J.N. Canongia Lopes, J. Deschamps, A.A.H. Padua

J Phys Chem B, 2004, 108, 11250-11250.

NA	14.00700	0.15000	1
CR	12.01100	-0.11000	1
NA	14.00700	0.15000	1
CW	12.01100	-0.13000	1
CW	12.01100	-0.13000	1
C1	12.01100	-0.17000	1
HCR	1.00800	0.21000	1
C1	12.01100	-0.17000	1
HCW	1.00800	0.21000	1
HCW	1.00800	0.21000	1
H1	1.00800	0.13000	1
H1	1.00800	0.13000	1
H1	1.00800	0.13000	1
C2	12.01100	0.01000	1
H1	1.00800	0.13000	1
H1	1.00800	0.13000	1
CS	12.01100	-0.12000	1
HC	1.00800	0.06000	1
HC	1.00800	0.06000	1
HC	1.00800	0.06000	1
HC	1.00800	0.06000	1
CS	12.01100	-0.12000	1
HC	1.00800	0.06000	1
HC	1.00800	0.06000	1

CS	12.01100	-0.12000	1
HC	1.00800	0.06000	1
HC	1.00800	0.06000	1
CS	12.01100	-0.12000	1
HC	1.00800	0.06000	1
HC	1.00800	0.06000	1
CT	12.01100	-0.18000	1
HC	1.00800	0.06000	1
HC	1.00800	0.06000	1
HC	1.00800	0.06000	1

ATOM	1	N1	0.000	0.000	0.000
ATOM	2	C2	0.000	0.000	1.315
ATOM	3	N3	1.237	0.000	1.760
ATOM	4	C4	2.082	0.000	0.672
ATOM	5	C5	1.311	0.000	-0.426
ATOM	6	C6	-1.180	-0.000	-0.870
ATOM	7	H7	-0.884	0.000	1.936
ATOM	8	C8	1.654	0.000	3.166
ATOM	9	H9	3.162	0.000	0.697
ATOM	10	H10	1.653	0.000	-1.450
ATOM	11	H11	-1.168	-0.890	-1.500
ATOM	12	H12	-2.082	-0.000	-0.259
ATOM	13	H13	-1.168	0.890	-1.500
ATOM	14	C14	2.857	0.926	3.342

ATOM	15	H15	0.831	0.351	3.789
ATOM	16	H16	1.928	-1.012	3.464
ATOM	17	C17	3.291	0.926	4.808
ATOM	18	H18	3.680	0.575	2.720
ATOM	19	H19	2.584	1.939	3.045
ATOM	20	H20	2.469	1.278	5.431
ATOM	21	H21	3.566	-0.085	5.106
ATOM	22	C22	4.503	1.859	4.985
ATOM	23	H23	5.318	1.501	4.391
ATOM	24	H24	4.241	2.848	4.672
ATOM	25	C25	4.920	1.884	6.467
ATOM	26	H26	5.173	0.893	6.783
ATOM	27	H27	4.108	2.250	7.060
ATOM	28	C28	6.141	2.806	6.642
ATOM	29	H29	6.954	2.438	6.052
ATOM	30	H30	5.888	3.796	6.324
ATOM	31	C31	6.555	2.834	8.125
ATOM	32	H32	6.805	1.844	8.445
ATOM	33	H33	5.742	3.204	8.716
ATOM	34	C34	7.777	3.755	8.301
ATOM	35	H35	8.064	3.774	9.331
ATOM	36	H36	8.590	3.386	7.711
ATOM	37	H37	7.526	4.745	7.982
